# Artificial Diets with Altered Levels of Sulfur Amino Acids Induce Anticancer Activity in Mice with Metastatic Colon Cancer, Ovarian Cancer and Renal Cell Carcinoma

**DOI:** 10.3390/ijms24054587

**Published:** 2023-02-27

**Authors:** Julio José Jiménez-Alonso, Emilio Guillén-Mancina, José Manuel Calderón-Montaño, Víctor Jiménez-González, Patricia Díaz-Ortega, Estefanía Burgos-Morón, Miguel López-Lázaro

**Affiliations:** Department of Pharmacology, Faculty of Pharmacy, University of Seville, 41012 Sevilla, Spain

**Keywords:** sulfur amino acids, anticancer activity, metastasis, cancer metabolism, colorectal cancer, ovarian cancer, renal cancer, methionine, cysteine, taurine, amino acids

## Abstract

Sulfur-containing amino acids methionine (Met), cysteine (Cys) and taurine (Tau) are common dietary constituents with important cellular roles. Met restriction is already known to exert in vivo anticancer activity. However, since Met is a precursor of Cys and Cys produces Tau, the role of Cys and Tau in the anticancer activity of Met-restricted diets is poorly understood. In this work, we screened the in vivo anticancer activity of several Met-deficient artificial diets supplemented with Cys, Tau or both. Diet B1 (6% casein, 2.5% leucine, 0.2% Cys and 1% lipids) and diet B2B (6% casein, 5% glutamine, 2.5% leucine, 0.2% Tau and 1% lipids) showed the highest activity and were selected for further studies. Both diets induced marked anticancer activity in two animal models of metastatic colon cancer, which were established by injecting CT26.WT murine colon cancer cells in the tail vein or peritoneum of immunocompetent BALB/cAnNRj mice. Diets B1 and B2B also increased survival of mice with disseminated ovarian cancer (intraperitoneal ID8 *Tp53^−/−^* cells in C57BL/6JRj mice) and renal cell carcinoma (intraperitoneal Renca cells in BALB/cAnNRj mice). The high activity of diet B1 in mice with metastatic colon cancer may be useful in colon cancer therapy.

## 1. Introduction

Cancer can often be cured by surgery when the tumor cells are localized to the primary site. However, when cancer cells disseminate and surgical resection of all metastatic tumors is not possible, cancer generally becomes an incurable disease. For example, although the 5-year relative survival rate of patients diagnosed with localized colon cancer is over 90%, this percentage drops to 15% for patients diagnosed with distant metastases [1]. Likewise, the 5-year survival rates of patients with disseminated renal cell carcinoma and ovarian cancer treated with the available therapies are 14% and 30%, respectively [1]. Many patients who survive five years after diagnosis eventually die of the disease. New therapies are necessary for patients with metastatic cancers. 

Cancer metabolism is emerging as a potential target for development of new anticancer therapies. Cancer cells develop a variety of metabolic alterations that allow them to survive and proliferate [2,3]. These alterations include changes in uptake and metabolism of glucose [4], amino acids (AAs) [5,6] and lipids [7] as well as changes in redox balance regulation [8]. The higher demand for specific essential AAs (EAAs) and non-essential AAs (NEAAs) is a key metabolic feature of cancer cells. For example, cancer cells often lack the ability to synthesize sufficient levels of NEAAs and need to take them from the extracellular environment [5]. Dietary and pharmacological restriction of specific AAs has already shown anticancer activity in different cancer types. For example, dietary restriction of methionine (Met) [9,10,11,12,13], cysteine (Cys) [13,14,15], serine–glycine [16,17,18,19,20,21] and arginine [22,23] has shown anticancer activity in preclinical colon cancer models. 

Although AA manipulation induces anticancer effects in a variety of preclinical cancer models, it is unclear which AAs offer greater potential for therapeutic intervention. A recent screening of 18 artificial diets in mice with disseminated renal cell carcinoma revealed that the effect of restricting particular AAs on the anticancer activity of the diets was much more complex than previously thought [24]. Previous studies suggested that the presence or absence of a particular AA is what determines the anticancer activity of strategies based on manipulating AAs. However, our in vivo screening showed that restriction of individual AAs could have a positive or negative effect on anticancer activity depending on levels of other AAs [24]. Since the anabolic and catabolic routes of most AAs are heavily interconnected, the requirement of specific AAs is probably influenced by the levels of other AAs. For example, most NEAAs can be biosynthesized from glucose and glutamate/glutamine. Glucose generates metabolites that provide carbon, hydrogen and oxygen atoms for most NEAAs, while glutamate provides their amino group; glutamate can directly or indirectly take the nitrogen atom from the amino group of any AA [25]. Glucose and glutamate, however, are insufficient for the biosynthesis of NEAA Cys. This AA needs a fifth type of atom, sulfur, that can only be provided by EAA Met. The sulfur atom in Met and Cys restricts their metabolic coupling with other AAs. Manipulating sulfur-containing AAs may, therefore, provide a less complex and more effective way of targeting AA metabolism for cancer therapy.

Sulfur-containing AAs Met and Cys play important biological roles. These two proteinogenic AAs are necessary not only for protein synthesis but also for other cellular processes [26]. For example, EAA Met is the precursor of S-adenosyl methionine, which is a methyl donor involved in DNA methylation and epigenetics [27]. Met also produces Cys through the irreversible transsulfuration pathway [28]. Cys is a precursor of several sulfur-containing molecules that play important cellular roles [29], including glutathione (GSH) [30,31], hydrogen sulfide (H_2_S) [32] and taurine (Tau) [33]. Tau is the most abundant free AA in animal tissues [26]. Although Tau is not used for protein synthesis, it has several important physiological functions, including neutralization of oxidative molecules and regulation of cellular osmolality [33,34,35,36,37]. 

Met restriction is known to exert in vivo anticancer effects [9,10,11,12,13,24,38,39,40,41,42]. However, since Met is a precursor of Cys and Tau, the anticancer activity of Met restriction may be mediated, at least in part, by restriction of Cys and Tau. In fact, Cys restriction is already known to induce anticancer activity [13,14,15,43]. Alternatively, since Cys and Tau have important biological roles, their restriction may cause non-selective toxicity and limit the anticancer activity of Met-deficient diets. In this work, we screened the in vivo anticancer activity of several Met-deficient diets supplemented with Cys, Tau or both to better understand the role of sulfur-containing AAs in cancer therapy and to try to develop more effective anticancer treatments. 

## 2. Results

### 2.1. Screening of Methionine-Deficient Diets Suplemented with Cysteine and Taurine in Mice with Ovarian Cancer

We have previously shown that an artificial diet deficient in Met, Cys and Tau (diet TC5, Table in Section 4.3) induced marked anticancer activity in mice with metastatic colon cancer; mice fed this diet lived longer than mice treated with standard anticancer drug capecitabine [13]. The same diet (also called diet T18) was also active in mice with renal cell carcinoma [24]. To evaluate if this diet was also active in ovarian cancer, we used an ovarian cancer model, which was established by inoculating murine ID8 *Trp53*^−/−^ ovarian cancer cells into the peritoneum of female C57BL/6JRj mice [44]. Treatments started 14 days after inoculation of the cancer cells. Anticancer drug cisplatin was used as a positive control; it was administered intraperitoneally once a week for 4 weeks at a dose of 5 mg/kg. Mice fed diet TC5 lived 7.5 days longer than untreated mice; survival (mean ± SEM; days) was 46.5 ± 1.0 for control mice (*n* = 4) and 54.0 ± 2.7 for mice treated with diet TC5 (*n* = 4); the *p*-value, calculated with the Gehan–Breslow–Wilcoxon test (GBW test), was 0.0601. Mice treated with cisplatin (*n* = 4) were sacrificed on day 67 with mild signs of disease; autopsy revealed small tumors in their peritoneum. This experiment showed that diet TC5 was active not only in mice with colon and renal cancers [13,24] but also in mice with ovarian cancer. However, anticancer drug cisplatin was much better than diet TC5 in this cancer model. Since cisplatin was administered in the peritoneal cavity, it may exert a direct cytotoxic effect on intraperitoneally disseminated cancer cells; this may contribute to explain its high anticancer activity.

We next used this animal model to screen the anticancer activity of six Met-deficient diets supplemented with Cys, Tau or both. These diets were based on diet TC5 (detailed composition in Table in Section 4.3). Instead of cisplatin, we used oral capecitabine as a positive control [45]. Since this is a highly reproducible cancer model, and to follow the recommendations of the Animal Ethics Committee, we used three mice in each group for screening. Table 1 shows that all diets improved mice survival and were better than several cycles of capecitabine. These data indicate that Met-deficient diets supplemented with Cys, Tau or both induced anticancer activity in mice with disseminated ovarian cancer. Diets B1 and B2B showed the highest anticancer activity and were selected for further studies.

**Figure 1:**
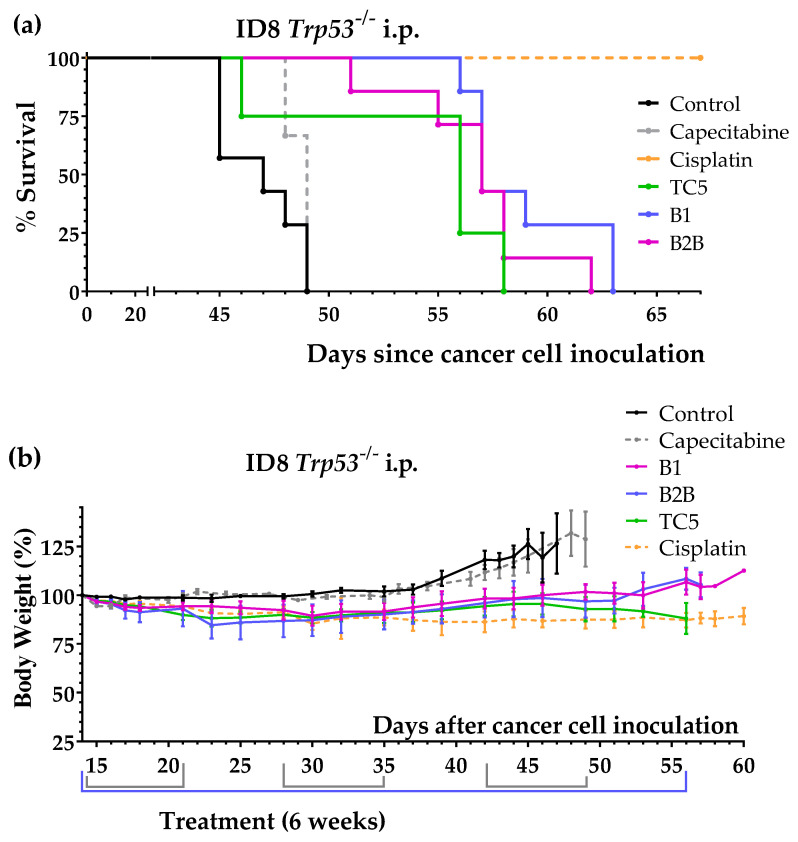
Anticancer activity of several artificial diets, capecitabine and cisplatin in mice with peritoneally disseminated ovarian cancer (ID8 *Trp53^−/−^* murine ovarian cancer cells inoculated in the peritoneum of immunocompetent C57BL/6JRj mice). (**a**) Survival of untreated mice (control), mice treated with oral capecitabine (450 mg/kg/day, 7/7 schedule, 3 cycles), mice treated with cisplatin (intraperitoneal administration of 5 mg/kg once a week for 4 weeks) and mice treated with diets TC5, B1 and B2B for 6 weeks (normal diet was replaced by one of these diets). (**b**) Body weights of the mice (mean percentage ± SEM) relative to body weights at the beginning of treatments (day 14).

An additional independent experiment confirmed the anticancer activity of diets B1 and B2B in this cancer model. The global survival (mean ± SEM) was 46.9 ± 0.7 days for control mice (*n* = 7), 58.9 ± 1.1 days for mice fed diet B1 (*n* = 7) and 56.9 ± 1.3 days for mice fed diet B2B (*n* = 7) (Figure 1a). The survival improvements achieved with both diets were statistically significant when compared with untreated mice (*p*-value < 0.001; GBW test). Diets B1 and B2B were very well tolerated, and body weights were not substantially reduced (Figure 1b). This additional experiment also showed that supplementing Met to diet B2B slightly reduced but did not abolish anticancer activity; survival of mice fed diet B2B+Met was 54.5 ± 1.6 days (*n* = 4). Figure 1 shows survivals and body weights of mice treated with diet TC5, diet B1, diet B2B, capecitabine and cisplatin.

### 2.2. Diets B1 and B2B Induce Moderate Anticancer Activity in Mice with Renal Cell Carcinoma 

**Figure 2:**
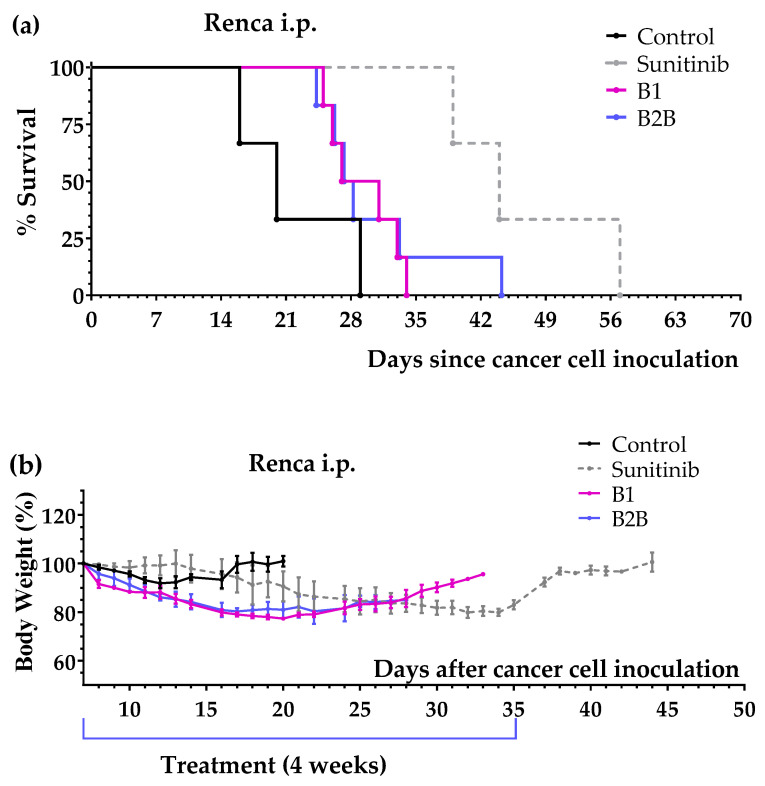
Anticancer activity of several artificial diets and sunitinib in mice with peritoneally disseminated renal cell carcinoma (Renca cells in BALB/c mice). (**a**) Survival of untreated mice (control), mice treated with oral sunitinib (60 mg/kg/day for 4 consecutive weeks) and mice treated with diets B1 and B2B for 4 weeks. (**b**) Body weights of mice (mean percentage ± SEM) relative to body weights at the beginning of the treatments (day 7).

The anticancer activity of B1 and B2B diets was further evaluated in mice with renal cell carcinoma. Treatments started 7 days after inoculation of Renca (murine renal cell carcinoma) cells into the peritoneum of immunocompetent BALB/cAnNRj mice. Sunitinib (60 mg/kg/day, oral administration), which is a first-line anticancer drug for patients with metastatic renal cell carcinoma, was used as a positive control. Figure 2a shows that mice treated with diets B1 and B2B lived several days longer than untreated mice. These diets, however, were less effective than sunitinib. The treatments were well tolerated, although moderate weight loss was observed (Figure 2b). Table 2 summarizes mice survival in each treatment group. 

### 2.3. Diets B1 and B2B Induce Marked Anticancer Activity in Mice with Metastatic Colon Cancer

We next evaluated the anticancer activity of diets B1 and B2B in two colon cancer models, which were established by injecting CT26.WT murine colon cancer cells in the tail vein (pulmonary metastases) or peritoneum (peritoneal dissemination) of immunocompetent BALB/cAnNRj mice. Capecitabine (450 mg/kg/day), a first-line treatment for patients with metastatic colon cancer, was also evaluated in these models. All treatments started four days after inoculation of the cancer cells.

**Figure 3:**
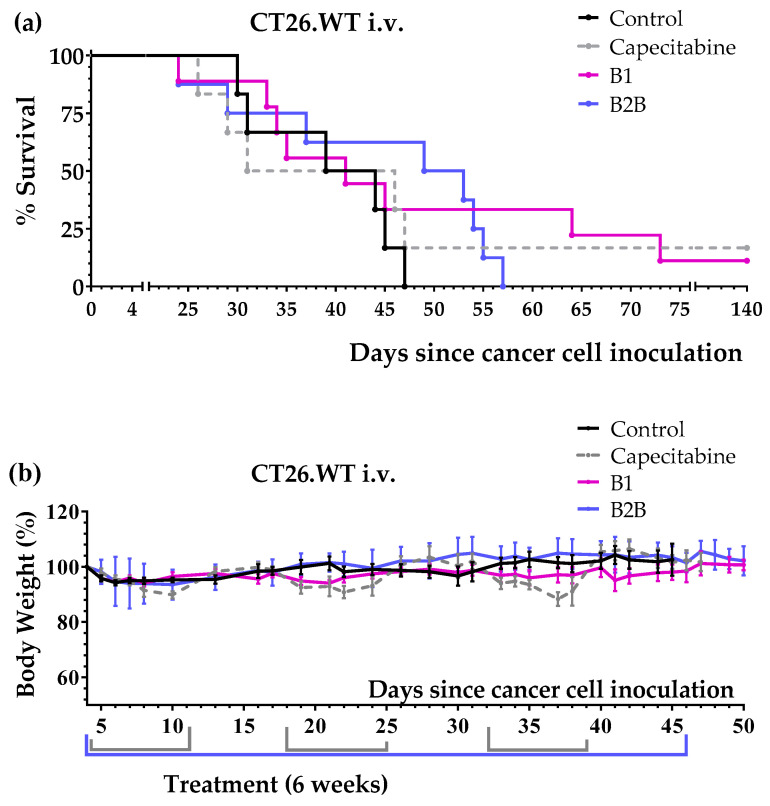
Anticancer activity of diet B1, diet B2B and capecitabine in mice with metastatic colon cancer (CT26.WT murine colon cancer cells inoculated in the tail vein of immunocompetent BALB/c mice; lung metastases model). (**a**) Survival of untreated mice (control), mice treated with oral capecitabine (450 mg/kg/day, 7/7 schedule, 2–3 cycles) and mice treated with diets B1 and B2B for 6 weeks. (**b**) Body weights of mice (mean percentage ± SEM) relative to body weights at the beginning of the treatments (day 4).

First, we evaluated diets B1 and B2B in the colon cancer model with pulmonary metastases. The results, shown in Figure 3 and Table 3, revealed that both diets improved mice survival. Diet B1 was more active than diet B2B. The activity of diet B1 was similar to that of capecitabine. One mouse treated with diet B1 and one mouse treated with capecitabine survived treatment; these mice were sacrificed on day 140 and no tumors were found on autopsy. Diets B1 and B2B were very well tolerated, and no significant weight losses were observed (Figure 3b). 

**Figure 4:**
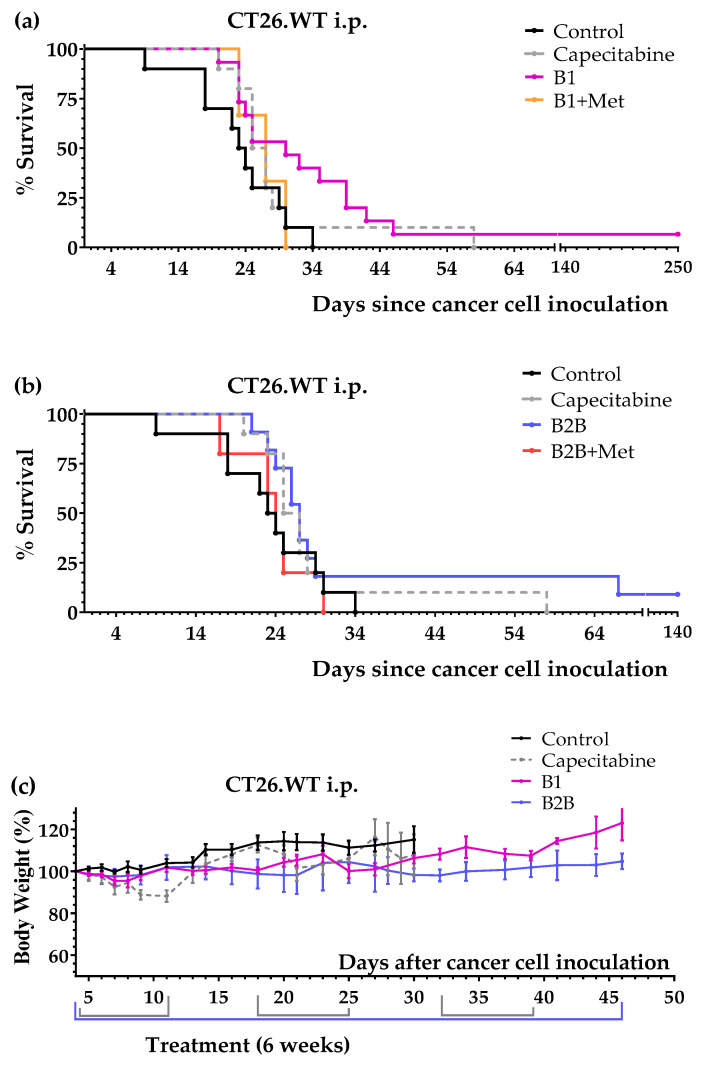
Anticancer effect of several artificial diets and capecitabine in mice with metastatic colon cancer (CT26.WT murine colon cancer cells inoculated in the peritoneum of immunocompetent BALB/c mice; peritoneal dissemination model). (**a**) Survival of untreated mice (control), mice treated with oral capecitabine (450 mg/kg/day, 7/7 schedule, 2–3 cycles) and mice treated with diets B1 and B1+Met for 6 weeks. (**b**) Survival of untreated mice (control), mice treated with oral capecitabine and mice treated with diets B2B and B2B+Met for 6 weeks. (**c**) Body weights of the mice (mean percentage ± SEM) relative to body weights at the beginning of treatments (day 4).

Next, we evaluated the anticancer activity of diets B1 and B2B in the intraperitoneal colon cancer model. Several mice were also treated with diet B1+Met and diet B2B+Met (detailed composition in Table in Section 4.3) to evaluate the effect of supplementing Met on the anticancer activity of the diets. The results, shown in Figure 4 and Table 4, indicate that diets B1 and B2B markedly improved survival of mice with colon cancer with peritoneal dissemination. Mice fed these diets lived longer than mice treated with several cycles of first-line drug capecitabine. One mouse fed diet B1 was sacrificed on day 250; the autopsy revealed the presence of a metastatic tumor in the lungs. One mouse fed diet B2B also had very long survival; it was sacrificed on day 250 and no tumors were found on autopsy. Diets B1 and B2B were very well tolerated, and no significant weight losses were observed. Mice treated with capecitabine showed signs of toxicity and suffered weight losses that reverted at the end of each treatment cycle (Figure 4c). Supplementing Met to diets B1 and B2B markedly reduced their anticancer activity, therefore indicating that Met restriction was important for the activity of these diets.

## 3. Discussion

Despite continuous development of new anticancer drugs, most patients with unresectable metastatic cancers do not overcome the disease. The aim of this work was to find more effective treatments for patients with metastatic cancers. Our approach was to exploit the genetic and metabolic defects of cancer cells as a whole by using artificial diets in which levels of AAs and other nutrients are strongly manipulated. These artificial diets would create unfavorable metabolic environments that would selectively kill cancer cells. Unlike cancer cells, normal cells have a functional genome and would adapt to the temporal nutrient imbalances created with the artificial diets [25]. Our recent results indicate that this strategy induces selective anticancer activity and improves the survival rates of mice with different types of metastatic cancers [13,24,46]. In this work, we sought to develop more effective artificial diets by manipulating the levels of sulfur-containing AAs Met, Cys and Tau.

We and others have already shown that Met restriction induces anticancer effects in mice with different cancer types. However, since Met is a precursor of Cys and Tau, the role of Cys and Tau in the anticancer activity of Met-restricted diets is unclear. For example, we recently showed that diet TC5 induced a marked anticancer effect in mice with colon cancer [13]. In this diet, 6% casein is the only source of sulfur-containing AAs, which provide low levels of Met (0.17%), very low levels of Cys (0.042%) and 0% Tau. In this and other Met-restricted diets, levels of Cys and Tau are also highly restricted or absent. Reducing Cys and Tau may have a positive effect on the anticancer activity of Met-deficient diets. For example, Met restriction can lead to Cys restriction, reduced GSH production and generation of cytotoxic levels of ROS in cancer cells. Since Cys produces H_2_S, Cys deprivation can also reduce levels of H_2_S, which may facilitate cancer cell elimination by the immune system [47]. However, if Met is directly responsible for the anticancer activity of Met-deficient diets, Cys and Tau restriction induced by these diets may have a negative effect on anticancer activity, for example by causing non-selective cytotoxicity or impairing immune function. In fact, both H_2_S and Tau have been shown to improve function of various immune cells [48,49]. If this is the case, supplementing Met-deficient diets with Cys and/or Tau may increase their anticancer activity.

We prepared six Met-deficient diets based on diet TC5; this diet induced anticancer activity in mice with metastatic colon cancer and renal cell carcinoma [13,24]. The diets were supplemented with Cys, Tau or both (Table in Section 4.3). All diets contained 1% lipids (salmon oil) because we have previously observed that reducing lipid levels can increase the anticancer activity of artificial diets based on AA manipulation [13,24,46]. To follow the recommendations of the Animal Ethics Committee and reduce the number of animals to a minimum, we selected a highly reproducible animal model of ovarian cancer to screen these diets. The results revealed that all Met-deficient diets increased survival of mice with disseminated ovarian cancer (Table 1). Diets B1 and B2B showed the highest activity. Mice fed these two diets lived several days longer than mice fed diet TC5 (Figure 1a). Unexpectedly, supplementing Met to one of these diets (diet B2B+Met) did not significantly reduce its anticancer activity, therefore suggesting that Met restriction was not necessary to achieve anticancer activity in this cancer model. The reduced lipid levels (1%) and casein levels (6%) in all these diets may contribute to explain their anticancer activity. The activity of the two most active diets, B1 and B2B, was confirmed in an independent experiment. Although the mechanism is unclear, these two diets significantly improved the survival of mice with disseminated ovarian cancer. Anticancer drug cisplatin (administered intraperitoneally) was better than any of the diets, while anticancer drug capecitabine was virtually ineffective in this cancer model. 

In mice with disseminated renal cell carcinoma, diets B1 and B2B also improved mice survival by several days. However, these diets were worse than first-line drug sunitinib (Figure 2 and Table 2). Diets B1 and B2B were also less effective than several related artificial diets, including diet TC5 [24].

Diets B1 and B2B improved mice survival in two animal models of metastatic colon cancer. Diet B1 was better than diet B2B in both cancer models. In the tail vein colon cancer model (pulmonary metastases), the activity of diet B1 was similar to that of first-line drug capecitabine (Figure 3 and Table 3). In the colon cancer model with peritoneal dissemination, mice fed diet B1 or B2B lived longer than mice treated with several cycles of capecitabine (Figure 4 and Table 4). Both diets were very well tolerated and induced lower toxic effects than capecitabine. The anticancer activity of both diets was strongly reduced when Met was supplemented (Figure 4 and Table 4), therefore indicating that Met restriction was important for the activity of these diets. Diet B1 induced high anticancer activity in both colon cancer models, which was comparable to that of diet TC5 [13].

Our previous and current data in mice with metastatic cancers suggest that replacing the normal diet of cancer patients with our artificial diets for several weeks may be therapeutically useful. Currently, an artificial diet based on diet TC5 (containing 6% casein, 5% Gln, 2.5% Leu and 1% salmon oil) is being evaluated as monotherapy in patients with different types of metastatic cancers. Although our artificial diets may be useful as monotherapy, it would also be important to evaluate their anticancer activity in combination with the standard drugs used in patients with specific types of cancer. If the survival rates achieved with the combination therapy were higher than with the standard drugs alone, it might become the new standard of care for patients with those specific types of cancer.

The role of sulfur-containing AAs in the anticancer activity of a diet is complex and seems to depend on the type of cancer and levels of other AAs. We previously showed that several diets with relatively high amounts of Met induced striking anticancer activity in mice with renal cell carcinoma; however, supplementing similar levels of Met to a different set of diets completely abolished their anticancer activity [24]. In this work, Met supplementation strongly reduced the anticancer activity of diets B1 and B2B in mice with colon cancer. In contrast, Met supplementation did not significantly reduce the activity of diet B2B in mice with ovarian cancer. A similar complexity is observed with Cys. This work shows that the presence or absence of Cys does not have a significant impact on the anticancer activity of several diets in mice with ovarian cancer (Table 1). However, our previous work in mice with renal cell carcinoma showed that Cys supplementation reduced the activity of our most active diet, while adding a Cys supplement to an inactive diet markedly increased its anticancer activity [24]. In addition, we previously showed that 0.2% Cys supplementation to diet TC5 markedly reduced its anticancer activity in mice with colon cancer [13], but this work shows that diet B1, which contains 0.2% Cys, induces marked anticancer activity in mice with colon cancer. Unlike diet TC5, diet B1 lacks the 5% glutamine supplement, therefore suggesting that the glutamine/Cys ratio may be important for activity of diets in mice with colon cancer. The influence of glutamine on Cys requirements might be mediated by cystine/glutamate antiporter xCT [50,51]. Altogether, our results strongly suggest that changing the levels of sulfur-containing AAs can have a positive, negative or neutral effect on the anticancer activity of a dietary intervention depending not only on type of cancer but also on levels of other AAs. 

## 4. Materials and Methods

### 4.1. Cell Lines and Cell Culture Conditions

Mouse CT26.WT colon cancer cells and mouse Renca renal cell carcinoma cells were obtained from the American Type Culture Collection (ATCC, Manassas, VA, USA). CT26.WT cell line was grown in RPMI 1640. Mouse ID8 *Trp53*^−/−^ ovarian cancer cells were a gift from Dr. Iain A. McNeish (Institute of Cancer Sciences, University of Glasgow, Glasgow, UK [44]) and were grown in Dulbecco’s modified Eagle’s medium (DMEM) high-glucose medium. All media were supplemented with 100 U/mL penicillin, 100 µg/mL streptomycin and 10% fetal bovine serum, except medium for ID8 *Trp53*^−/−^, which was supplemented with 0.11g/l sodium pyruvate, 4% FBS and 1% insulin-transferrin-selenium. All cells were kept at 37 °C in a humidified atmosphere containing 5% CO_2_. Cell culture reagents were purchased from Biowest (Nuaillé, France) or Thermo Fisher Scientific (Waltham, MA, USA).

### 4.2. Drugs and Reagents

L-cystine (A1703), L-leucine (A1426) and L-methionine (A1340) were obtained from Panreac Química S.L.U. (Barcelona, Spain). Casein (isolated from bovine milk, 276070010), taurine (166541000) choline bitartrate (450225000), tert-butylhydroquinone (150822500), sunitinib malate (462640010) and cisplatin were purchased from Thermo Scientific Acros Organics (Waltham, MA, USA). Mineral Mix (AIN-93M-MX, 960401) and Vitamin Mix (AIN Vitamin Mixture 76, 905454) were acquired from MP Biomedicals (Eschwege, Germany). Sucrose was purchased from a local market (MAS Supermarket, Seville, Spain). Cellulose and corn starch were bought from Farmusal (local pharmacy, Granada, Spain). The lipid source of the diets was salmon oil (marketable oil developed for Pets Purest, Wilmslow, England, B06WWFTRXM). L-glutamine was obtained from Myprotein (Manchester, England). Capecitabine (500 mg/tablet, Normon, local Pharmacy, Seville, Spain) and India Ink (Superblack India Ink, Speedball, 33X089A, Greenville, CA, USA) were also used in this study. 

### 4.3. Diets Preparation and Composition

The diets were prepared by mixing all solid ingredients until they formed a well-blended dry powder. Next, oil and water were added to the mixture to make a soft dough. The dough was air-dried for about 2 h, manually pelleted (approximately 5 g/pellet), air-dried for an additional 24 h and stored until use. When Cys was added to diets, it was in the form of cystine (dimer of Cys). Table 5 shows the composition of the artificial diets used in this work. 

Casein (Thermo Scientific Acros Organics; 27607; bovine casein) was used as a protein source. The typical amount (g) of AAs in 100 g and 6 g (shown in brackets) of the casein used in the experiments was glutamine + glutamate: 21.7 (1.302), leucine: 9 (0.54), methionine: 2.9 (0.174), phenylalanine: 4.8 (0.288), histidine: 2.6 (0.156), lysine: 7.5 (0.45), threonine: 4.1 (0.246), isoleucine: 4.3 (0.258), valine: 5.3 (0.318), tryptophan: 1.2 (0.072), cysteine/cystine: 0.7 (0.042), arginine: 3.4 (0.204), glycine: 1.7 (0.102), serine: 5.7 (0.342), tyrosine: 5.2 (0.312), alanine: 2.9 (0.174), aspartate + asparagine: 6.9 (0.414) and proline: 10.1 (0.606).

Mineral Mix (AIN-93M-MX, MP Biomedicals) constituted 3.5% of the dry diet. 100 g of diet contained (g) calcium carbonate (1.25), monopotassium phosphate (0.875), potassium citrate (0.098), sodium chloride (0.259), potassium sulfate (0.163), magnesium oxide (0.085), ferric citrate (0.021), zinc carbonate (0.0058), manganese carbonate (0.0022), copper carbonate (0.0011), potassium iodate (0.000035), sodium selenate (0.000035), ammonium paramolybdate-tetrahydrate (0.000028), sodium metasilicate-nonahydrate (0.0051), chromium potassium sulfate-dodecahydrate (0.00095), lithium chloride (0.0000595), boric acid (0.000284), sodium fluoride (0.00022), nickel carbonate hydroxide (0.00011), ammonium meta-vanadate (0.000021) and sucrose (0.73).

Vitamin Mix (AIN Vitamin Mixture 76, MP Biomedicals) made up 1% of the dry diet. 100 g of dry diet contained (mg) thiamine hydrochloride (0.6), riboflavin (0.6), pyridoxine hydrochloride (0.7), nicotinic acid (3), D-calcium pantothenate (1.6), folic acid (0.2), D-biotin (0.02), cyanocobalamin (0.001), retinyl palmitate premix (250,000 IU/g) (1.6), DL-a-tocopherol acetate (250 IU/g) (20), cholecalciferol (400,000 IU/g) (0.25), menaquinone (0.005) and sucrose (972.9).

The control diet used in this study was the ssniff diet (SM R/M-S E, 10 mm; V1724-000; Soest, Germany). The composition, expressed as crude materials, is 21% protein, 7% fat, 4% fiber, 6.2% ash, 33.3% starch and 4.6% sugar. The control diet contained 0.91% Met and 0.38% Cys in its composition. 

### 4.4. Animals

Female BALB/cAnNRj mice, male BALB/cAnNRj mice and female C57BL/6JRj (10 weeks or older) were obtained from Janvier Labs® (Le Genest-Saint-Isle, France). To enable adequate acclimation, the animals were kept in our animal laboratory facilities for at least two weeks before the experiments started. The mice were kept in standard conditions (12 h light/12 h dark cycle, 70–75% humidity, 24 °C, with ad libitum access to food and water). The animals were fed a standard diet (ssniff diet R/M-Z E/R/S; V1724-000, ssniff Spezialdiäten). All mice were 12 weeks or older at the start of the experiments.

The experiments were approved by the Animal Ethics Committee of the University of Seville (CEEA-US2018-6/2 and CEEA-US2019-20) and Junta de Andalucía (15/05/2018/090 and 13/11/2020/131) and were carried out following the recommendations of the European Union on animal experimentation (Directive of the European Counsel 2010/630/EU). To follow the recommendations of the Animal Ethics Committee and reduce the number of mice to a minimum, treatments were initially screened using 3–4 mice per group. Additional independent experiments were carried out to confirm the anticancer effect of the active diets. Mice receiving the same treatment are merged in the survival curves to facilitate comparison between groups. The number of animals used for each treatment is shown in the text and/or tables in the Results section. All experiments included a control group (untreated mice) and a positive control group.

### 4.5. In Vivo Cancer Models

Cell processing for all the in vivo cancer models used in this work was similar. Murine cells (5th–7th passage) were cultured in 75 cm^2^ flask until approximately 60–70% confluence. Then, the medium was removed and the cells were washed twice with sterile PBS. The cells were then incubated with a trypsin/EDTA solution for 3 min at 37 ° C. Next, cells were detached and resuspended in 5 mL of sterile PBS and the cell suspension was pipetted up and down to break up any cell aggregate before adding 2.5%-FBS-supplemented medium. Then, a working cell suspension (between 5 × 10^5^ and 25 × 10^6^ cells/mL depending on the cancer model) was prepared. The cell suspension was centrifuged (250 g) at room temperature for 5 min. The medium was then removed, and cells were resuspended in warm sterile-filtered PBS. Cells were counted again to ensure that the cell suspension was at the correct density. Finally, a 1 mL syringe (insulin type with a 29-G × 1/2″needle, BD, Madrid, Spain) was filled with 0.2 mL of the working cell suspension, which was injected into the peritoneal cavity or in the tail vein of the mice [13]. 

Mice were housed in individual cages one day before starting the treatments. Treatments started 4, 7 or 14 days (depending on the model) after injecting the cancer cells. The untreated animals (control group) continued to be fed a standard diet (ssniff diet). In the positive control group, mice were treated with a standard anticancer drug used in cancer patients. In the groups of mice treated with the artificial diets, the treatment simply consisted of replacing their normal diet with an artificial diet (see Table 5) in which the levels of specific AAs and lipids were manipulated. 

The ovarian cancer model (peritoneal dissemination) was established by inoculating 5 × 10^6^ ID8 *Trp53^−/−^* cells into the peritoneal cavity of female C57BL/6JRj mice [44]. Treatments started 2 weeks after cancer cell inoculation. Cisplatin or capecitabine were used as positive controls. Cisplatin (5 mg/kg) was administered intraperitoneally once a week for 4 weeks. Capecitabine was administered orally with the standard diet at an estimated dose of 450 mg/kg/day following a 7-day on, 7-day off schedule (7/7) to maximize the anticancer activity of this anticancer drug [52]. Mice were treated with the artificial diets for 6 consecutive weeks, and the mice that survived that period returned to the standard diet.

The renal cell carcinoma model (peritoneal dissemination) was established by inoculating 1.5 × 10^5^ Renca cells into the peritoneal cavity of male BALB/cAnNRj mice. Treatments started 7 days after cancer cell inoculation. Positive control sunitinib was administered daily in the diet at a dose of approximately 60 mg/kg/day for 4 weeks. Mice were treated with the artificial diets for 4 weeks.

The colon cancer models were established by inoculating 10^5^ CT26.WT cells into the peritoneal cavity (peritoneal dissemination model) or in the tail vein (lung metastasis model) of female BALB/cAnNRj mice [13,53,54]. In both models, treatments started 4 days after cancer cell inoculation due to the aggressiveness of these cancer models [13,54]. Capecitabine, which is a first-line drug for patients with metastatic colon cancer, was used at positive control. It was administered orally with the standard diet at an estimated dose of 450 mg/kg/day following a 7-day on, 7-day off schedule (7/7) [52]. Mice were treated with the artificial diets for 6 consecutive weeks and then returned to the standard diet. 

All animals were monitored daily, and body weights were recorded periodically (at least three times per week). Mice were sacrificed by cervical dislocation when signs of cancer progression were evident, such as respiratory distress, reduced curiosity and mobility and excessive losses or gains in body weight. These signs indicated that survival for additional 2 days was unlikely. Necropsy was performed to verify cause of death and observe the extent of the disease. Autopsies confirmed presence of tumors and similar tumor loads in all euthanized mice (unless otherwise specified) [13]. 

### 4.6. Statistical Analysis

Data are expressed as mean values ± standard error of the mean (SEM). Statistical analysis was performed with GraphPad Prism version 7.0 software. Statistical analysis for the Kaplan–Meier survival curves was calculated using the GBW test. 

## 5. Conclusions

In this work, we have explored the in vivo anticancer activity of several Met-deficient diets supplemented with Cys, Tau or both. Two of these diets were evaluated in mice with several types of metastatic cancers. Diets B1 and B2B showed anticancer activity in mice with disseminated colon cancer, ovarian cancer and renal cell carcinoma. The diets were very well tolerated. Diet B1 (6% casein, 2.5% leucine, 0.2% Cys and 1% lipids) showed marked activity in two animal models of metastatic colon cancer. Mice fed diet B1 as monotherapy lived longer than mice treated with several cycles of first-line anticancer drug capecitabine. Met supplementation blocked the anticancer activity of diets B1 and B2B, therefore indicating that Met restriction was crucial for the activity of the diets in colon cancer. This Met dependency was not observed in ovarian cancer. The role of sulfur-containing AAs in the anticancer activity of artificial diets seems to depend on the type of cancer and levels of other AAs. Future studies are needed to better understand the role of sulfur AAs in the anticancer activity of artificial diets. The high anticancer activity of diet B1 in two animal models of metastatic colon cancer suggests that this diet may be useful in patients with metastatic colon cancer.

## 6. Patents

J.J. Jiménez-Alonso, E. Guillén-Mancina, J.M. Calderón-Montaño, V. Jiménez-González, E. Burgos-Morón and M. López-Lázaro are inventors of a patent related to this research licensed to AMINOVITA, S.L. and University of Seville.

## Figures and Tables

**Table 1:** Survival of mice with ovarian cancer treated with artificial diets or capecitabine.

Treatment	Survival Time (*n* = 3; Days)			Survival Time (Mean ± SEM; Days)	Survival Improvement vs. Control (Days)	*p*-Value vs. Control
Control	49	45	48	47.3 ± 1.2	0.0	-
Capecitabine	48	49	49	48.7 ± 0.3	+1.3	0.3458
Diet B1	56	59	63	59.3 ± 2.0	+12.0	0.0339
Diet B1A	57	55	57	56.3 ± 0.7	+9.0	0.0339
Diet B1B	57	56	58	57.0 ± 0.6	+9.7	0.0339
Diet B2	57	52	57	55.3 ± 1.7	+8.0	0.0339
Diet B2A	51	62	55	56.0 ± 3.2	+8.7	0.0339
Diet B2B	62	58	55	58.3 ± 2.0	+11.0	0.0339

*p*-value was calculated with the GBW test.

**Table 2:** Survival of mice with peritoneally disseminated renal cell carcinoma treated with sunitinib and diets B1 and B2B.

Treatment	*n*	Survival Time (Mean ± SEM; Days)	Survival Improvement vs. Control (Days)	*p*-Value vs. Control
Control	3	21.7 ± 3.8	-	-
Sunitinib	3	46.7 ± 5.4	+25.0	0.0339
B1	6	29.3 ± 1.6	+7.7	0.0833
B2B	6	30.3 ± 3.0	+8.7	0.1573

*p*-value was calculated with the GBW test.

**Table 3:** Survival of mice with metastatic colon cancer (lung metastasis model) treated with diet B1, diet B2B and capecitabine.

Treatment	*n*	Survival Time (Mean ± SEM; Days)	Survival Improvement vs. Control (Days)	*p*-Value vs. Control
Control	6	39.3 ± 3.0	-	-
Capecitabine	6	53.2 ± 17.7	+13.8	1.0000
B1	9	54.3 ± 11.9	+15.0	0.5964
B2B	8	44.8 ± 4.6	+5.4	0.2914

*p*-value was calculated with the GBW test.

**Table 4:** Survival of mice with metastatic colon cancer (peritoneal dissemination model) treated with capecitabine and several artificial diets.

Treatment	*n*	Survival Time (Mean ± SEM; days)	Survival Improvement vs. Control (days)	*p*-Value vs. Control
Control	10	23.2 ± 2.3	-	-
Capecitabine	10	28.8 ± 3.4	+5.6	0.2068
B1	15	45.1 ± 14.8	+21.9	0.0279
B1+Met	3	26.7 ± 2.0	+3.5	0.4300
B2B	11	39.8 ± 10.7	+16.6	0.1418
B2B+Met	5	23.8 ± 2.1	+0.6	0.9040

*p*-value was calculated with the GBW test.

**Table 5:** Composition of the artificial diets used in this work (g/100 g diet).

DIET	TC5	B1	B1A	B1B	B2	B2A	B2B	B1 + Met	B2B + Met
Casein	6.0	6.0	6.0	6.0	6.0	6.0	6.0	6.0	6.0
Glutamine	5.0	-	-	-	5.0	5.0	5.0	-	5.0
Leucine	2.5	2.5	2.5	2.5	2.5	2.5	2.5	2.5	2.5
Cystine	-	0.2	0.2	-	0.2	0.2	-	0.2	-
Taurine	-	-	0.2	0.2	-	0.2	0.2	-	0.2
Methionine	-	-	-	-	-	-	-	0.5	0.5
Salmon oil	1.0	1.0	1.0	1.0	1.0	1.0	1.0	1.0	1.0
Choline	0.25	0.25	0.25	0.25	0.25	0.25	0.25	0.25	0.25
Vitamin Mix	1.0	1.0	1.0	1.0	1.0	1.0	1.0	1.0	1.0
Mineral Mix	3.5	3.5	3.5	3.5	3.5	3.5	3.5	3.5	3.5
Sucrose	15.0	15.0	15.0	15.0	15.0	15.0	15.0	15.0	15.0
Cellulose	5.0	5.0	5.0	5.0	5.0	5.0	5.0	5.0	5.0
Corn starch	65.55	64.95	60.55	60.05	60.75	65.55	64.95	60.55	60.05
Tert-butylhydroquinone	0.0008	0.0008	0.0008	0.0008	0.0008	0.0008	0.0008	0.0008	0.0008
Total (g or %)	100	100	100	100	100	100	100	100	100

## Data Availability

Not applicable.
